# Small businesses, potentially large impacts: The role of fertilizer traders as agricultural extension agents in Bangladesh

**DOI:** 10.1108/JADEE-08-2017-0078

**Published:** 2019-05-15

**Authors:** Khondoker Abdul Mottaleb, Dil Bahadur Rahut, Olaf Erenstein

**Affiliations:** Socioeconomics Program, International Maize and Wheat Improvement Center (CIMMYT), El Batan, Mexico

**Keywords:** Production, Efficiency, Rural areas, Farmer, *Boro* rice, Fertilizers, Trader

## Abstract

**Purpose:**

Constraints associated with public agricultural extension services imply that farmers increasingly rely on input providers for agricultural innovations and knowledge. Yet such providers are typically commercial profit-making agents and may have an incentive to suggest relatively costly inputs and/or high rates. The purpose of this paper is to look into the case of Bangladesh and the role of fertilizer traders in terms of farmers’ decisions on which fertilizer to apply and at what rate. Using primary data, the authors examine farmers’ chemical fertilizer use and the associated rice production efficiency, based on different information sources (fertilizer traders, government extension agents or own/peer experience).

**Design/methodology/approach:**

Using primary data, the present study estimates an ordered probit model and production functions separately based on whether or not a farmer relied on information from fertilizer traders or own experience and government extension agents, and examines the efficiency score of each type of farmer.

**Findings:**

The findings demonstrate that the resource-poor farmers rely more on traders’ suggestions for fertilizer application than public extension – but the actual fertilizer information source has no significant effect on the production efficiency of the rice farmers. This study, therefore, does not find exploitative behavior of fertilizer traders. Thus, this study concludes that small rural traders in Bangladesh are working as agricultural extension agents and provide necessary fertilizer application information to resource-poor farmers.

**Research limitations/implications:**

This is a case study based on Bangladesh – an emerging economy in South Asia. The findings of the study may not be generalized for other countries.

**Originality/value:**

To the authors’ knowledge, this is the first study that confirms the role of agricultural input sellers as the extension agent in developing countries.

## 1 Introduction

The role of traders in reducing search and transaction costs and in disseminating useful market information is widely recognized, both in farm (e.g. Banerji and Meenakshi, 2004; Miyata *et al*., 2009) and non-farm sectors (e.g. Sonobe *et al*., 2002; Mottaleb and Sonobe, 2011). Further hindered by constraints associated with public agricultural extension services, farmers increasingly also rely on traders, such as input providers, for agricultural innovations and knowledge. Yet, such agricultural input traders are typically commercial profit-making agents and, aided by information asymmetry, may have an incentive to suggest relatively costly inputs and/or high rates, potentially exploiting farmers. For example, chemical fertilizer traders, who supposedly are knowledgeable about fertilizers, might prompt farmers to purchase and apply unnecessarily high fertilizer rates. Without easily verifiable quality assurances, some traders may even be tempted to supply adulterated and/or fake products. Such information asymmetry and possible exploitation by traders may increase agricultural production costs and inefficiency.

The potential exploitation of farmers by traders can be viewed as a principal-agent problem. Yet a strong social relationship and trust generated through repeated transactions between the farmers (principal) and the traders (agent) can mitigate the potential cheating behavior of traders (Arrow, 1968; Otsuka and Hayami, 1988). The importance of traders as well as social interactions for mutually beneficial economic outcomes for both traders and farmers in poverty-stricken developing countries has been studied variously (e.g. Fafchamps and Minten, 1999; Conley and Udry, 2010). Empirical studies, however, seldom explore the role of traders as agricultural extension agents in developing countries, and the outcome of such services on the production efficiency of resource-poor farmers. It is important to examine the issue, as reportedly overall agricultural production costs have been increasing, whereas the profitability and the overall well-being, particularly of smallholders, have been decreasing (e.g. Mottaleb and Mohanty, 2015).

**Figure 1:**
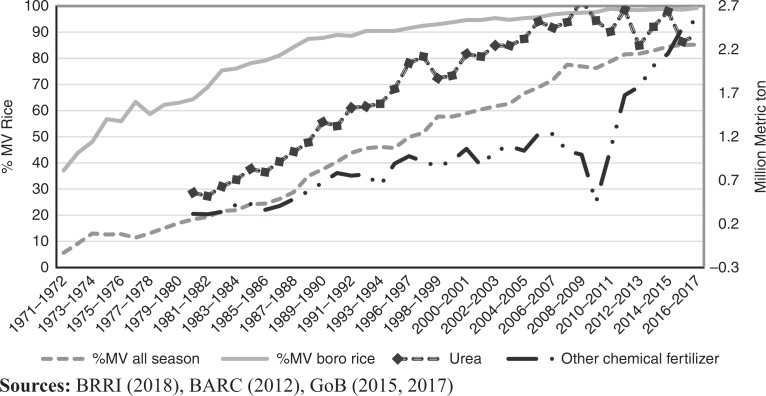
Modern variety adoption (% MV) by rice season (1971–2017) and fertilizer use (million metric ton, urea and other chemical fertilizer, 1981–2017) in Bangladesh

Using chemical fertilizer application by farm households in Bangladesh as a case, the present study examines the influence of traders as information source for farmers’ chemical fertilizer use and their production efficiency. In Bangladesh, chemical fertilizer use started in the 1960s associated with the dissemination of high-yielding modern varieties (MV) of rice, starting with IR8 (BARC, 2012) followed by a range of other varieties and more recently hybrid rice. The adoption of MV rice is particularly prominent in the irrigated *boro* season (mid-November to June), aided by the dramatic expansion of irrigation in Bangladesh in the 1970s (Figure 1). Chemical fertilizer use has long been emphasized by urea (as a source of nitrogen (N), followed by other chemical fertilizers to supply phosphorous (P), potassium (K) and sulfur (S) in various formulations.

For example, in 1981–1982 when 15 percent of the total land area was under MV rice (BRRI, 2018), the total chemical fertilizer use in Bangladesh was 0.88m tons, of which 64 percent (0.56m tons) was urea (BARC, 2012). In 2016–2017, MV rice covered 85 percent of the total rice area. Across seasons, it was even higher in the irrigated *boro* season at 99.2 percent. The other prominent seasons are the wet seasons of *aus* and *aman*. The total chemical fertilizer used was 4.91m tons of which 48 percent (2.37m tons) was urea (GoB, 2017).

Despite a long and wide experience in using chemical fertilizers (especially resource-poor) farmers in Bangladesh may still fail to use fertilizer efficiently and may incur losses. Modern crop varieties are particularly responsive to fertilizers, but to enhance the returns for their use, knowledge of chemical fertilizer use is indispensable, particularly the doses, types and the timing of applications (Islam, 2015). Farmers in Bangladesh depend on diverse information sources for such knowledge in addition to their own experience. Government agricultural extension officers, mass media and non-governmental organizations are the major formal information sources, complemented by fertilizer traders, neighboring farmers and farmers’ own experience as major informal sources (GoB, 2003). The Bangladesh Government has a Department of Agricultural Extension (DAE) which consists of 26,042 workers, including 14,090 sub-assistant agricultural officers (SAAOs) (Perveen, 2016) to provide extension services (BBS, 2017). However, with 15.18m farm households, this amounts to more than 1,000 farm households per sub-assistant agricultural office. Therefore, most farmers often need to rely on their own experience, and or peer experience, or on alternative providers for fertilizer use suggestions. Due to the constraints in accessing formal information sources (e.g. Rivera *et al*., 2001) and the easy access to traders, farmers increasingly may rely on traders’ suggestions to decide on fertilizer applications. This study examines the role of fertilizer traders on fertilizer use and the corresponding production efficiency for *boro* rice farmers in Bangladesh using primary data.

The case is worth investigating for several reasons. Bangladesh is one of the most densely populated countries in the world, with more than 1,077 person per square kilometer (GoB, 2017). Due to the enormous population pressure, the per capita arable land has been declining sharply since the 1960s. For example, in 1961, the per capita arable land in Bangladesh was 0.17 ha, but by 2013 the figure had been reduced to 0.05 ha only (World Bank, 2017). At present, the average farm size in the country is 0.69 ha (Rapsomanikis, 2015). As there is little or no scope to expand the land frontier to produce more crops to meet the increasing food demand, farmers in Bangladesh continuously intensify their agricultural production system (Ahmed, 1995).

Chemical fertilizers are a major input for agricultural intensification. Bangladesh produces fertilizer to meet the growing demand, although it increasingly relies on imports to fill in the supply–demand gap. For example, in 2005–2006, the country produced 51 percent (1.93m tons) of its chemical fertilizers use, but in 2013–2014, it only produced 22 percent (0.97m tons) (GoB, 2015). At present, BCIC (2015) operates six urea fertilizer factories[1], one triple super phosphate (TSP) factory and one di-ammonium phosphate (DAP) factory. To ensure self-sufficiency in food production, the government encourages farmers to use chemical fertilizers by providing fertilizer subsidies. For example, in 2014–2015, the government subsidy amounted to $865m for urea and other fertilizers, up by 67.5 percent over the $517m in 2008–2009 (MoA, 2017)[2].

Although Bangladesh Chemical Industries Corporation (BCIC) is solely responsible for fertilizer production and the Bangladesh Agricultural Development Corporation (BADC) ensures import, both BCIC and BADC jointly ensure storage, supply and distribution of fertilizers using their own channels. By November 2013, there were 8,437 BCIC/BADC-approved fertilizer dealers/wholesalers and 26,394 active fertilizer retailers operating in 490 sub-districts (DAE, 2015). The fertilizer prices are also fixed by the government at the dealer and retailer levels. For example, in the 2015–2016 fiscal year, the dealer urea price was set at Bangladesh Taka (BDT) T14/kg, TSP BDT20/kg, DAP BDT23/kg, muriate of potash (MoP) BDT13/kg and gypsum BDT10/kg, and at the retailer level, farmers had to pay extra BDT2/kg for each type of fertilizer.

The major objective of this study is to examine the impact of the source of fertilizer information on fertilizer use and production efficiency in Bangladesh, particularly the role of its fertilizer traders. This study thereby, provides the first empirical evidence on the role of traders on the fertilizer use and production efficiency of farmers. The rest of the study is organized as follows: Section 2 describes the sampling process, the data used and the descriptive findings; in Section 3, we develop a conceptual framework and specify econometric models; Section 4 provides the econometric findings; and in Section 5 conclusions and recommendations are presented.

## 2 Materials, methods and descriptive findings

**Figure 2:**
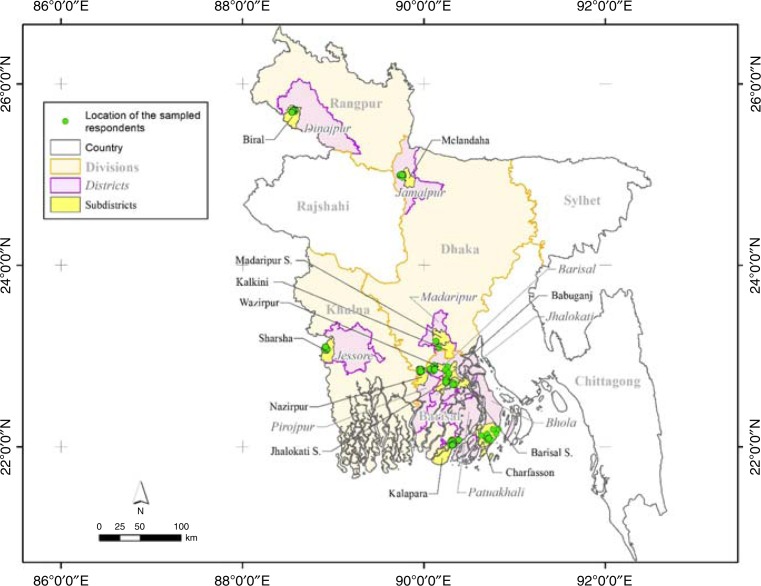
Location of the sampled households by sub-districts

The present study is based on primary data collected from 556 randomly selected farm households located in Barisal, Dhaka, Khulna and Rangpur divisions, including 9 districts, 12 sub-districts, 15 unions and 43 villages (Figure 2). From April 22 to June 8, 2015, the International Maize and Wheat Improvement Center (CIMMYT), Bangladesh, conducted a survey focusing primarily on collecting information from farm households on their crop production, application of chemical fertilizers and irrigation in the *boro* rice seasons in 2012–2013 and 2013–2014. As we have collected data by seasons from the same sampled households, the data are panel in nature. Of the four divisions, focus was on the Barisal Division in South-central Bangladesh, with five of its districts included in the sample against one to two districts for the other divisions. Compared to the national average, Barisal Division has a lower cropping intensity, defined as the number of crops grown per calendar year in a specific land area (MoA and FAO, 2013). This study, therefore, may reveal the heterogeneity in fertilizer application behavior of the farmers located in northern and southern regions of Bangladesh.

A multistage random sampling process was followed in selecting samples. In the first stage, the sampled (sub-)districts were selected based on the criterion that the selected (sub-) districts were equipped with the highest numbers of irrigation service providers (using BADC, 2013). Using the same criterion, we then selected unions and villages in the sampled (sub-)districts based on information provided by the SAAO, the field-level government extension office. From each sampled sub-district, we selected 1 union and 1–4 sample villages, and 4–16 farmers from each sampled village. The high-yielding *boro* rice in Bangladesh is cultivated from December to mid-February to mid-April to June (BBS, 2017). Farm and production data were collected in the first half of 2015, including recall data for the previous 2012–2013 and 2013–2014 seasons. The sampled households, on average, applied 309 kg of urea fertilizer and 268 kg other chemical fertilizer per hectare and spent BDT10,236/ha on all types of chemical fertilizers (Table I). Considering the fact that the nitrogen content in urea in Bangladesh is 46 percent, the sampled farmers in Bangladesh applied 142 kg of nitrogen/ha in the periods surveyed.

**Table I:** Number of sampled households and the application of urea and other chemical fertilizer (kg/ha) by location of the households

Division	District	Sub-district	No. of sampled households	Urea applied (kg/ha)	Other chemical fertilizer (kg/ha)	Total expenditure on all chemical fertilizer (BDT/ha)
Barisal	Barisal	Babuganj	8	380.4	310.4	12,285
		Barisal Sadar	16	286.9	219.8	9,026
		Wazirpur	76	313.7	285.8	10,651
	Bhola	Char Fasson	64	293.9	327.2	9,664
	Jhalokati	Jhalokati Sadar	64	318.9	227.2	9,509
	Patuakhali	Kalapara	64	141.3	133.9	4,934
	Pirojpur	Nazirpur	64	385.8	299.7	12,301
Dhaka	Jamalpur	Melandaha	64	342.4	287.5	11,030
	Madaripur	Kalkini	4	268.5	224.0	8,585
		Madaripur	4	369.9	198.6	9,754
		Sadar			
Khulna	Jessore	Sharsha	64	393.9	335.1	13,062
Rangpur	Dinajpur	Birol	64	293.9	327.2	10,839
Total/average			556	309.1	268.1	10,236

**Source:** Authors’ survey (2015)

Table II presents information on input use and yields for *boro* rice, two season’s average, in Bangladesh by the farmers’ source of information. Out of 556 sampled farm households, 32 percent decided their fertilizer use following the suggestion of the fertilizer traders, 58 percent relied on their own experience or on the suggestions of the neighbor famers (peer experience) and only 10 percent relied on the suggestions provided by the public extension agents. On average, the sampled farmers cultivated *boro* rice on 0.22 ha and applied 309 kg of urea and 268 kg of other chemical fertilizers per hectare. The other chemical fertilizers mainly include DAP, TSP, MoP and gypsum. On average, a sampled farmer applied 703 kg of compost (farm yard and poultry manure), applied 178 labor-days and used 150 kg of seeds per hectare. Nearly 32 percent of the sampled farmers cultivated hybrid rice, and the average rice yield was 6.44 tons/ha (Table II).

**Table II:** Farmer-reported input use and yields for *boro* rice by source of information, average 2012–2013 and 2013–2014 for study area locations Bangladesh

	Source of information				
	All	Fertilizer trader	Own/peer experience	Government extension agent	*F*-statistic^a^
No. of observations	556	175	324	57	
Sample share (% hh)	100	31.5	58.3	10.2	
*Boro* rice area (ha)	0.22 (0.18)	0.22 (0.14)	0.22 (0.21)	0.20 (0.15)	0.51 (0.60)
Urea (kg/ha)	309 (206)	300 (161)	303 (219)	372 (237)	5.98*** (0.00)
Other fertilizer (DAP, TSP, MoP, gypsum, kg/ha)	268 (166)	279 (144)	263 (184)	266 (113)	1.06 (0.35)
Compost (kg/ha)	703 (2,483)	580 (2,416)	808 (2,686)	481 (1,067)	1.47 (0.23)
Labor (man-day/ha)	178 (87)	183 (89)	173 (85)	187 (92)	2.18 (0.11)
Seed (kg/ha)	150 (189)	147 (201)	157 (195)	122 (86)	1.77 (0.17)
Hybrid seed (% hh)	31.6 (46.5)	30.9 (46.3)	32.7 (47.0)	27.2 (44.7)	0.74 (0.48)
Yield (t/ha)	6.44 (2.98)	6.59 (2.65)	6.32 (3.04)	6.63 (3.34)	1.19 (0.30)

**Notes:** Numbers in parentheses are standard deviations, except

a Prob. > *F* values, with *F* values in parentheses. *H*_0_: Mean (a) = Mean (b) = Mean (c).

*** Indicates 1 percent level of significance

**Source:** Survey (2015)

Farmers who relied on government extension agents applied more urea fertilizer (372 kg/ha) on average than the other farmers (Table II), whereas farmers who relied on fertilizer traders or their own experience/peers applied a similar 300 kg/ha. The use of other fertilizers showed no significant difference among the three groups, nor were there significant differences in other input use or reported yields. This suggests that the rural fertilizer traders in Bangladesh: are not cheating farmers by advising excessive use rates nor providing fake and adulterated products; and are providing an adequate substitute source of information to the farmers similar to the government extension agents. Hybrid rice farmers applied more chemical fertilizers than non-hybrid users. On average, a hybrid rice sampled farmer applied 340 kg of urea and 302 kg of other chemical fertilizers per hectare, whereas non-hybrid rice farmers applied a significantly lower 295 kg of urea and 252 kg of other chemical fertilizers.

Table III presents selected background information of the sampled households. On average, a sampled household head is more likely to be male (97 percent of the cases), 45 years old, with 4.6 years of schooling and with nearly five family members, at least two of which are directly engaged in, or provide support to, the family’s agricultural work (including the household head). Nearly 50 percent of the sampled households reported that at least one of their family members was a member of 21 listed clubs, organization or groups (including farmers’ clubs, women’s unions, youth unions, NGOs providing micro-credit or agricultural extension services, any other NGOs, formal or informal credit groups, environmental groups, school and mosque committees, traders’ unions, labor unions and any other type of village association). Nearly 41 percent of the sampled households had at least one blood relative (including brother/sister or first cousin) who is politically active or a government employee (in agriculture or any other sector). In our econometric model, we considered these variables as the proxies for social capital of the sampled households.

**Table III:** Basic information of sampled farm households by source of information and study area locations Bangladesh

	Source of information				
	All	Fertilizer trader	Own/peer experience	Government extension agent	F-statistic^a^
No. of observations	556	175	324	57	
Sample share (% hh)		31.5	58.3	10.2	
Age, household head (years)	45.4 (12.9)	44.9 (13.0)	45.3 (12.9)	47.5 (12.7)	1.71 (0.18)
% Female-headed household	3.1 (83.9)	2.0 (87.1)	4.1 (80.0)	1.8 (87.8)	2.39* (0.09)
Years of schooling, household head	4.59 (4.62)	4.42 (4.52)	4.39 (4.35)	6.30 (5.99)	8 77*** (0.00)
Total no. of family members	4.69 (1.55)	4.83 (1.45)	4.61 (1.62)	4.65 (1.42)	2.41* (0.09)
No. of family members engaged in agriculture (extend support or full time)	2.06 (1.07)	2.01 (1.07)	2.10 (1.10)	1.95 (0.89)	1.56 (0.21)
Member of club or other organizations (% hh)	43.7 (49.7)	40.6 (49.2)	44.1 (49.7)	50.9 (50.4)	1.39 (0.24)
At least one blood relative in a government job or politics (% hh)	40.7 (49.2)	41.1 (49.4)	38.6 (48.8)	50.9 (50.4)	3.07** (0.5)
Farm size (ha, cultivated in 2013–2014)	0.83 (1.11)	0.76 (0.57)	0.88 (1.35)	0.79 (0.83)	1.39 (0.24)
*Boro* rice area (ha, 2013–2014)	0.22 (0.18)	0.22 (0.14)	0.22 (0.21)	0.20 (0.16)	0.51 (0.60)
Distance from the household to nearest market (km)	1.73 (1.18)	1.73 (1.15)	1.75 (1.13)	1.70 (1.52)	0.09 (0.91)
No. of markets within 5 km radius	1.74 (2.10)	1.95 (2.76)	1.42 (1.06)	2.88 (3.40)	27.0*** (0.00)
No. of power tillers in the village	6.06 (4.11)	5.79 (3.94)	6.23 (4.19)	5.86 (4.24)	1.48 (0.22)
Village connected to electricity grid (% villages)	54.1 (49.9)	55.4 (49.9)	50.9 (50.1)	68.4 (46.9)	6.20*** (0.00)
Cumulative length of paved/gravel road at the village level (km)	5.60 (11.41)	9.17 (16.50)	3.90 (7.68)	4.28 (6.75)	26.23*** (0.00)
Adequate irrigation water availability in the *boro* season (% hh)	76.1 (42.7)	82.3 (38.3)	74.4 (43.7)	66.7 (47.6)	7.06*** (0.00)

**Notes:** Numbers in parentheses are standard deviations, except

a Prob. > *F* values, with *F* values in parentheses. *H*_0_: Mean (a) = Mean (b) = Mean (c).

*,**,*** Indicate the 10, 5 and 1 percent levels of significance, respectively

**Source:** Survey (2015)

On average, a sampled household cultivated 0.83 ha of land in 2013–2014, of which 0.22 ha (26.5 percent) was under *boro* rice, with the cultivated area a proxy for wealth. On average, a sampled household is located 1.73 km from the closest market, and there are nearly two markets within a 5-km radius of the location of a sampled household – both used as a proxy for the availability of traders and social relationships with traders as households located closer to a market might meet traders more often than others in many social occasions in addition to market transactions of agricultural inputs. The availability of traders as well as social relations with traders may greatly influence whether or not a household will rely on traders’ suggestions for fertilizer applications in their crop fields and it may influence the quality of suggestions a trader may provide to a household.

On average, there were at least six power tillers (two-wheel tractors) in a sampled village, used as a proxy for the state of agricultural mechanization in a village. Over half (54 percent) of the sampled villages were connected to the electricity grid, and each sampled village had 5.6 km of paved or gravel road (Table III). These physical infrastructure indicators have been used as proxies for the overall access to agricultural information by households in a village as both formal and informal information sources might be more available in villages with good-to-excellent physical infrastructures creating easy access, as opposed to other villages with poor infrastructures. Three-quarters of the sampled households reportedly had adequate irrigation water during the 2013–2014 *boro* season. We use this information to capture the influence of the bio-physical environment on fertilizer use.

Table III also disaggregates the background information of the sampled households by the sources of information households relied on for fertilizer use. Household heads who relied on the government extension officers as sources of information were more educated than those who relied on other sources, suggesting relatively well-educated households may have easier access to government information, as educated household heads can more easily read government leaflets and booklets and can communicate with the agricultural extension officers with more confidence than others. Households relying on traders as a source of information tended to have larger families, whereas female-headed households were more likely to rely on their own/peer experience. Interestingly, households with a blood relative in a government job or politics were more likely than others to rely on government information. These findings suggest that relatively resource-poor farm households (in terms of human and social capital) are more likely to rely on the suggestions of fertilizer traders or their own/peer experience. Households relying on government extension as information sources had more markets within a 5-km radius from a sampled village and were more likely connected to the electricity grid. In contrast, households relying on traders as information sources reported a larger cumulative length of road at the village level and less frequent irrigation water shortages (Table III).

In Table III, the contrasts among the sampled households based on the source of information they relied on do not control for the potentially confounding effects of other influential variables. To econometrically differentiate the factors associated with differences in fertilizer use among the sampled households based on the source of information they relied on, we developed a conceptual framework and empirical models in the next section.

## 3 Conceptual framework and model specifications

Farmers in Bangladesh depend on both informal and formal sources of information to decide on fertilizer application. Among the sampled farmers, only 57 (10 percent) relied on formal sources of information from government extension agents on deciding fertilizer use. In contrast, 31.5 percent relied on traders and the bulk (58 percent) relied on their own/peer experience. Suggestions from government extension agents are completely free of monetary costs and supposedly objective and of higher quality than suggestions from other sources. A farmer’s own/peer experience is ground-truthed in the local context and generally without monetary costs. A trader may provide new information, and thereby act as a *de facto* extension agent, which is particularly useful where farmers lack access to formal information sources. Still, reliance on suggestions from traders might entail hidden costs based on information asymmetry, whereby a trader might induce farmers to apply more fertilizers than warranted just to maximize the traders’ own profits.

To characterize households based on their information source for fertilizer use, we developed the following equation including household head’s characteristics (*i*), household-specific characteristics (*h*) and village (*v*) and sub-district-level (*d*) characteristics:

$${\text{Y}}_{ij}=f(A_{i},FHD_{i},SCL_{i},FIA_{i},SN_{h},FS_{h},VA_{h},FHD_{i},VC_{v},YD_{y},SDD_{d})+\varepsilon_{i},$$ (1)

where Y_*ij*_ is a vector of dependent variables that assumes the value 1 if a household relied on traders, assumes the value 2 if a household relied on their own/peer experience and assumes the value 3 if a household relied on the government extension agents’ suggestion to decide on fertilizer use; *A_i_* is the age of the household head; *FHD_i_* is a dummy that assumes a value of 1 if a household head is female (0 otherwise), *SCL_i_* is the years of schooling of the household head; *FIA_i_* is the total number of family members engaged in agriculture (either full time or occasionally); *SN_h_* is the social network dummy that assumes a value of 1, if at least one of its members is a member of listed village-level farmers’ club or any village-level organization, or 0 otherwise; *FS_h_* is the farm’s *boro* rice area (ha); *VA_h_* is a variety dummy that assumes a value of 1, if a household used hybrid rice seed, and 0, otherwise; *VC_v_* is a vector of variables including village-level physical infrastructure and characteristics: distance from the household to the nearest market (km), the number of markets within a 5-km radius, the number of power tillers in the village, the cumulative length of paved or gravel road and a water availability dummy that assumes a value 1 if the household reported no water scarcity for irrigating *boro* rice in the 2012–2013 and 2013–2014 seasons, or 0 otherwise; *YD_y_* is a dummy for the season 2013–2014 (yes = 1), where the base is the 2012–2013 season. *SDD_d_* includes 11 dummies for 12 sampled sub-districts to capture the sub-district level of unobserved influences affecting a household’s decision to rely on available sources for suggestions on fertilizer use, where Babuganj, a sub-district of Barisal District of Barisal Division, is the base (assigned a value 0).

The dependent variable is categorical and is ordered based on the quality of services: the traders’ suggestion is treated as “potentially biased” (potentially against farmers’ interests), farmers’ own/peer experience is treated as “subjective standard” (farmers’ current self-interests) and the suggestion of the government extension agents is treated as “objective improvement” (potentially in farmers’ interests). To estimate Equation (1), we applied an ordered probit estimation method.

This study further examined the production efficiency of the sampled farmers to assess the impacts of the sources of information on fertilizer use. In order to examine the level of technical efficiency in a way consistent with the theory of production function, this study first specified a Cobb–Douglas type stochastic frontier production function as follows:

$$Q_{it}=\beta_{0}+\sum_{i=1}^{k}\beta_{it}X_{it}+V_{it}-U_{it},$$ (2)

and:

$$Q_{it}=\beta_{0}+\sum_{i=1}^{5}\beta_{it}X_{it}+\sum_{i=1}^{7}\sum_{i=1}^{7}\beta_{ijt}X_{ijt}+V_{it}-U_{it},$$ (3)

where in Equations (2) and (3) *Q_i_* is the rice yield (kg/ha); *X*_1_ the seed used (kg/ha); *X*_2_ the urea fertilizer (kg/ha); *X*_3_ the other fertilizer (kg/ha); *X*_4_ the compost (kg/ha); *X*_5_ the labor applied (labor day/ha); *U_i_* is the farmer-specific production efficiency; and *V_i_* the statistical disturbance term, assumed to be independently and identically distributed, having $N(0,\sigma_{v}^{2})$ distribution pattern. The efficiency scores are estimated as follows: *TE_it_* = *E*[exp (−*u_i_*|ε_*i*_)], where *TE_it_* is the technical efficiency score of the individual farmer *i* in season *t*.

Following Greene (2005), a true random-effects model estimation approach was applied to estimate Equation (3), using the procedure suggested by Belotti *et al*. (2013). In estimating Equation (3), the distribution of the error term is assumed to be exponential, which is set as the default in the econometrics programming.

## 4 Econometric findings

Table IV presents estimated functions applying an ordered probit estimation method, explaining sources of information that a farm household in Bangladesh relied on to decide fertilizer use. Female-headed households are more likely to rely on their own/peer experience in deciding fertilizer use. Sampled households with strong social networks, proxied by organizational/club membership, were less likely to depend on suggestions from traders. Neither the *boro* rice area nor the use of hybrid rice influenced the sources of information used. Interestingly, remoteness, as proxied by the distance from the nearest market, increased reliance on government extension agents and decreased dependence on traders for fertilizer advice. This likely reflects that closer to the market, traders are more available and, thus, more likely to provide suggestions, and vice versa. The level of agricultural mechanization, as proxied by the number of power tiller owners in the village, reduced dependence on government extension agents in deciding fertilizer use, which is probably associated with the availability of irrigated land and market integration. Similarly, cumulative road length at the village level increased dependence on traders (who can more easily and frequently visit such villages) and reduced dependence on own/peer experience and government extension agents as information sources. However, households located in villages connected to the electricity grid are less likely to depend on traders, but are more likely to depend on their own/peer experience and government extension agents for deciding on fertilizer use. An electricity connection can enhance the information inflow because electronic and other mass media provide agriculture and fertilizer-related information and broadcast bulletins on agriculture. Households with adequate irrigation water are more likely to depend on traders and less likely to depend on their own/peer experience and government extension agents for deciding on fertilizer use. This reflects the association between water availability and *boro* rice cultivation, with *boro* rice cultivation being relatively intensive and market oriented. Households in Barisal Sadar (Barisal District) and Kalkini (Madaripur District) are less likely to rely on traders’ suggestions for fertilizer use, compared to the base Babuganj (Barisal District). At present, a number of NGOs and other organizations are actively working in Barisal on agricultural development, as it is lagging behind than other regions in terms of agriculture.

**Table IV:** Estimated functions applying the ordered probit model estimation procedure explaining sources of fertilizer information used by farmers in their *boro* rice fields, study area locations Bangladesh

Estimation method		Marginal effects		
Dependent variable: source of information	Ordered probit	Y = Pr (source = fertilizer trader)	Y = Pr (source = own/peer experience)	Y = Pr (source = government extension agent)
Age, household head	0.01 (0.004)	0.002 (0.001)	0.001 (0.001)	0.001 (0.001)
Female-headed household (dummy, yes = 1)	0.32 (0.27)	−0.10 (0.08)	0.04** (0.02)	0.06 (0.06)
Years of schooling, household head	0.01 (0.01)	−0.004 (0.004)	0.003 (0.002)	0.002 (0.002)
No. of family members engaged in agriculture (extend support or full time)	−0.02 (0.05)	0.01 (0.02)	−0.003 (0.01)	−0.002 (0.01)
Member of club or some other organizations (dummy, yes = 1)	0.21* (0.11)	−0.07 (0.04)	0.04* (0.02)	0.03* (0.02)
*Boro* rice area (ha)	−0.28 (0.28)	0.10 (0.10)	−0.05 (0.05)	−0.04 (0.04)
Hybrid rice (dummy, yes = 1)	−0.12 (0.13)	0.04 (0.05)	−0.02 (0.03)	−0.02 (0.02)
Distance from household to the nearest market (km)	0.13** (0.06)	−0.05** (0.02)	0.03 (0.01)	0.02** (0.01)
No. of markets within 5-km radius	−0.03 (0.05)	0.01 (0.02)	−0.01 (0.01)	−0.01 (0.01)
No. of power tillers in the village	−0.03* (0.018)	0.01 (0.01)	−0.01 (0.003)	−0.01* (0.003)
Village connected to electricity grid (dummy, yes = 1)	0.37*** (0.13)	−0.13*** (0.05)	0.07*** (0.03)	0.06*** (0.02)
Cumulative length of paved/gravel road at the village level (km)	−0.05*** (0.01)	0.02*** (0.003)	−0.01*** (0.002)	−0.01*** (0.001)
Adequate irrigation water availability (dummy, yes = 1)	−0.28** (0.12)	0.09** (0.04)	−0.05*** (0.02)	−0.05** (0.02)
Season 2013–2014 (dummy, base season 2012–2013)	−0.003 (0.003)	0.001 (0.001)	−0.001 (0.001)	−0.001 (0.0004)
*Sub-districts (dummies, base = Babuganj)*				
Barisal Sadar	1.25* (0.69)	−0.27*** (0.07)	−0.08 (0.20)	0.36 (0.25)
Birol	−0.54 (0.59)	0.20 (0.23)	−0.14 (0.19)	−0.06 (0.05)
Char Fasson	0.80 (0.66)	−0.22 (0.15)	0.04 (0.06)	0.18 (0.20)
Jhalokati Sadar	−0.20 (0.60)	0.07 (0.22)	−0.05 (0.15)	−0.03 (0.07)
Kalkini	1.208 (1.02)	−0.26*** (0.10)	−0.08 (0.30)	0.35 (039)
Madaripur Sadar	−0.45 (0.71)	0.17 (0.28)	−0.12 (0.23)	−0.05 (0.06)
Melandaha	0.0001 (0.60)	−0.00003 (0.21)	0.00001 (0.12)	0.00001 (0.09)
Nazirpur	−0.25 (0.59)	0.09 (0.22)	−0.06 (0.15)	−0.03 (0.07)
Sharsha	−0.21 (0.60)	0.08 (0.22)	−0.05 (0.15)	−0.03 (0.07)
Wazirpur	−0.16 (0.59)	0.06 (0.22)	−0.03 (0.14)	−0.02 (0.08)
Kalapara	−0.13 (0.61)	0.05 (0.22)	−0.03 (0.14)	−0.02 (0.08)
cut1	−0.95 (0.72)			
cut2	0.95 (0.72)			
No. of observations	1,112			
Wald χ^2^ (25)/*F*	74.10			
Probability > χ^2^	0.00			
Pseudo *R*^2^	0.07			
Log pseudolikelihood	−940.92			
				

**Notes:** Numbers in parentheses are standard errors calculated based on robust standard errors clustered at the household level.

*,**,*** Indicate the 10, 5 and 1 percent levels of significance, respectively

The parameters of the translog production frontier model (Equation (3)) are presented in Table V. The slope coefficients of the stochastic frontier describe the output elasticities of inputs and the estimated signs of the parameters are as expected. A 1 percent increase in seed and urea use increases *boro* rice yield by 0.47 and 1.29 percent, respectively (Table V). Other fertilizer (i.e. non-urea) use also increases yields: a 1 percent increase in use increased rice yield by 0.29 percent, with a further quadratic increase of 0.02 percent if use is doubled. A doubling of the compost dose can increase yield by 0.01 percent on average. Surprisingly, labor use is negatively related to rice yield: a 1 percent increase in labor-day use reduced rice yield by 1.12 percent with a further quadratic reduction of 0.15 percent if labor use is doubled. The interaction terms among the five inputs present consistent results. The interaction between seed and other fertilizer, seed and compost and seed and labor are all positive and increase rice yield, although seed and urea interaction is negative and reduces rice yields (at the current levels of application). The sub-district dummies show rice yields to be higher than the base sub-district Babuganj (Barisal District) for a number of study areas.

**Table V:** Parameter estimates generated by rice production function specifications applying time-invariant feasible generalized least square random-effects estimation procedure (dependent variable = ln(rice yield, kg/ha)), study area locations Bangladesh

	Parameter estimates	SE
ln(seed)	0.47***	0.07
ln(non-urea)	0.29***	0.05
ln(urea)	1.29***	0.49
ln(compost)	−0.02	0.04
ln(labor days)	−1.12**	0.49
ln(seed) × ln(seed)	−0.02	0.02
ln(non-urea) × ln(non-urea)	0.02***	0.003
ln(urea) × ln(urea)	0.0003	0.004
ln(compost) × ln(compost)	0.01***	0.002
ln(labor days) × ln(labor days)	−0.15*	0.08
ln(seed) × ln(non-urea)	0.07**	0.03
ln(seed) × ln(urea)	−0.14***	0.03
ln(seed) × ln(compost)	0.02***	0.01
ln(seed) × ln(labor days)	0.22**	0.09
ln(non-urea) × ln(urea)	−0.30***	0.06
ln(non-urea) × ln(compost)	0.01***	0.002
ln(non-urea) × ln(labor days)	0.24***	0.05
ln(labor days) × ln(compost)	−0.02***	0.01
Season 2013–2014 (dummy)	−0.05	0.04
*Sub-district dummies (base: Babuganj sub-district)*		
Barisal Sadar (dummy)	0.40*	0.24
Birol (dummy)	0.18	0.21
Char Fasson (dummy)	0.50**	0.21
Jhalokati Sadar (dummy)	0.40*	0.21
Kalkini (dummy)	0.62*	0.34
Madaripur Sadar (dummy)	0.21	0.34
Melandaha (dummy)	0.23	0.21
Nazirpur (dummy)	0.40*	0.21
Sharsha (dummy)	0.30	0.21
Wazirpur (dummy)	0.55***	0.21
Kalapara (dummy)	0.23	0.22
Constant	3.928***	0.53
Number of observations	1,112	
Σ*u*	0.26	
Σ*ν*	0.68	

**Notes:** Numbers in parentheses are standard errors.

*,**,*** Indicate the 10, 5 and 1 percent levels of significance, respectively

Finally, Table VI presents the efficiency scores of the sampled rice farmers by the information source they relied on to decide fertilizer use. On average, the sampled rice farmers in Bangladesh are estimated to be 71.9 percent efficient, which means there is still a possibility of enhancing rice production efficiency by nearly 28 percent. Importantly, the efficiency score is the highest for the farmers who relied on traders for their suggestions on chemical fertilizer application (72.5 percent), followed by the farmers who relied on their own or peer experience (71.8 percent). The efficiency score is the lowest for the farmers who relied on the government extension officers for suggestions on fertilizer application, which is 70.9 percent. The pairwise comparison of the mean efficiency score reveals that the farmers who relied on traders’ suggestion are statistically significantly more efficient by 0.70 percent than the farmers who relied on their own/peer experience (10 percent level) and by 1.60 percent than the farmers who relied on government extension agents (5 percent level). However, the difference in the efficiency score between the farmers who relied on their own/peer experience and the government extension agents is not statistically significant.

**Table VI:** Summary of technical efficiency scores by sources of information farmers relied on for deciding chemical fertilizer application

Source on information relied on							
	All	Trader 1	Own/peer experience 2	Government extension agents 3	Pairwise comparisons of mean efficiency score (unequal variances) by the sources of information relied on		
	1 vs 2	1 vs 3	2 vs 3
Technical efficiency score (%)	71.9	72.5	71.8	70.9	0.70* (0.39)	1.60** (0.72)	0.90 (0.70)

**Notes:** Values in parentheses are standard errors.

*,** Indicate the 10 and 5 percent levels of significance, respectively

**Source:** Authors’ calculation

## 5 Conclusion and policy recommendations

In developing countries, the emerging private sector is gradually filling in the gap between supply and demand of agricultural extension services (Feder *et al*., 2011). In Bangladesh, most farmers still use their own/peer experience, but increasingly seek suggestions from traders when deciding on the amount and dose of fertilizer to be applied due to the constraints associated with public agricultural extension services. Still, social and human capital and remoteness increase the dependence on government extension agents for fertilizer use decisions, whereas traders increasingly prevail as information sources in the more accessible, intensive and commercially oriented *boro* rice production systems. Fertilizer traders in Bangladesh thereby provide necessary information to the farmers to decide on fertilizer use. Although traders are profit-making agents, our findings indicate no evidence that traders exploit the farmers by encouraging them to apply more fertilizers compared to farmers who rely on their own/peer experience or government extension agents for these decisions. In addition, the production efficiency of the farmers who relied on traders is statistically higher than others. Thus, fertilizer traders in Bangladesh are in fact supplementing the agricultural extension works of the government by providing useful information to resource-poor farmers, which contributes to mitigating market failures and achieving higher production efficiency.

Expanding general education in developing countries is likely to be beneficial for agricultural development, as education enhances information processing abilities in general; however, returning farmers to school may not be a practical option. Alternatively, there may be scope for more agricultural training on improved and novel crop management (including fertilizer use) at the village level, particularly targeting female-headed farm households, in which local fertilizer traders could be directly involved. With the help of international donor agencies and local grass-roots organizations, the national government can enable the use of farmers’ clubs and to ensure and strengthen the training on fertilizers and related agricultural issues. Additionally, the government agricultural extension services may include local fertilizer traders as major stakeholders, and traders should be sensitized on the latest market information, particularly information on crop-based recommended fertilizer doses. Government agencies such as BCIC, and DAE using their own networks and support from international donor agencies should take the appropriate measures to sensitize retailers, as well as farmers on recommended fertilizer use. Local print and electronic media, including community radio, can be used to sensitize both farmers and traders to fertilizer use by season and crops.

Last but not least, there is scope for an increased role for the omnipresent small-scale fertilizer traders in Bangladesh and elsewhere. Local retailers are not only fertilizer sellers, but they also act as a source of information on which farmers rely and provide a widespread coverage of the rural landscape. Beyond being important agricultural input providers, they are creating even bigger positive social impacts by correcting possible market failures, and serving as a conduit for new agricultural innovations and knowledge, and thus contributing in achieving higher production efficiency. Such a complementary role as agricultural extension agent should be increasingly incorporated and integrated into farmers’ awareness and agricultural development programs.

## Notes

1 According to BCIC (2015), the urea fertilizer factory located in Fenchuganj, Sylhet, also produces ammonium sulfate.

2 The original fertilizer subsidy information was given in Bangladesh Taka (BDT), converted with an exchange rate BDT82 = $1.

## References

[cit0001] Ahmed R. (1995), “Liberalization of agricultural input markets in Bangladesh: process, impact and lessons”, *Agricultural Economics*, Vol. 12 No. 2, pp. 115–128.

[cit0002] Arrow K.J. (1968), “The economics of moral hazard: further comment”, *American Economic Review*, Vol. 58 No. 3, pp. 537–539.

[cit0003] BADC (2013), “Minor irrigation survey report 2012–13”, Bangladesh Agricultural Development Corporation, Ministry of Agriculture, Government of Bangladesh, Dhaka, June.

[cit0004] Banerji A. and Meenakshi J.V. (2004), “Millers, commission agents and collusion in grain auction markets: evidence from basmati auctions in north India”, Working Paper No. 129, Centre for Development Economics, Delhi School of Economics, New Delhi, available at: www.cdedse.org/pdf/work129.pdf (accessed November 26, 2015).

[cit0005] BARC (2012), *Fertilizer Recommendation Guideline – 2012*, Bangladesh Agricultural Research Council, Ministry of Agriculture, Dhaka, available at: www.moa.gov.bd/site/page/38479282-7a23-417f-8818-e7066013c699/%E0%A6%B8%E0%A6%BE%E0%A6%B0-%20%E0%A6%B8%E0%A7%81%E0%A6%AA%E0%A6%BE%E0%A6%B0%E0%A6%BF%E0%A6%B6-%E0%A6%A8%E0%A6%BF%E0%A6%B0%E0%A7%8D%E0%A6%A6%E0%A7%87%E0%A6%B6%E0%A6%BF%E0%A6%95%E0%A6%BE (accessed November 13, 2015).

[cit0006] BBS (2017), *Yearbook of Agricultural Statistics – 2016*, 28th Series, Bangladesh Bureau of Statistics, Statistics and Informatics Division, Ministry of Planning, Government of Bangladesh, Dhaka.

[cit0007] BCIC (2015), “Annual report 2014–15”, Bangladesh Chemical Industries Corporation, Ministry of Industries, Dhaka, July 6, available at: http://bcic.gov.bd/site/page/cf916677-6902-422e-bfb3-77327e27a20c/Financial-Year-of-2014-15 (accessed November 13, 2015).

[cit0008] Belotti F., Daidone S., Ilardi G. and Atella V. (2013), “Stochastic frontier analysis using STATA”, *Stata Journal*, Vol. 13 No. 4, pp. 719–758.

[cit0009] BRRI (2018), *Total Rice Area (000’ ha) in Bangladesh. Bangladesh Rice Knowledge Bank*, Bangladesh Rice Research Institute, Gazipur, available at: http://brri.portal.gov.bd/sites/default/files/files/brri.portal.gov.bd/page/b6531346_66a5_45e79cb0_d00c7c7009dc/Rice%20Area%20in%20Bangladesh_21.01.2018.pdf (accessed February 13, 2018).

[cit0010] Conley T.G. and Udry C.R. (2010), “Learning about a new technology: pineapple in Ghana”, *American Economic Review*, Vol. 100 No. 1, pp. 35–69.

[cit0011] DAE (2015), *Fertilizer Statistics: District Based BCIC Fertilizer Dealers and Retailers*, Department of Agricultural Extension, Ministry of Agriculture, Dhaka, available at: http://dae.portal.gov.bd/sites/default/files/files/dae.portal.gov.bd/page/1b881968_9bbe_4227_8878_fe351fe96e25/Fertilizer_Dealer%20list_14.05.2014.pdf (accessed November 13, 2015).

[cit0012] Fafchamps M. and Minten B. (1999), “Relationships and traders in Madagascar”, *Journal of Development Studies*, Vol. 35 No. 6, pp. 1–35.

[cit0013] Feder G., Birner R. and Anderson J.R. (2011), “The private sector’s role in agricultural extension systems: potential and limitations”, *Journal of Agribusiness in Developing and Emerging Economies*, Vol. 1 No. 1, pp. 31–54.

[cit0014] GoB (2003), *Agricultural Extension in Bangladesh: An Entitlement of All Farmers? The Result of a National Extension Coverage Survey*, Agricultural Services and Innovation Project, Department of Agricultural Extension, Ministry of Agriculture, Government of Bangladesh, Dhaka, available at: www.lcgbangladesh.org/Agriculture/reports/The%20Results%20of%20a%20National%20Extension%20Coverage%20Survey.pdf (accessed November 26, 2015).

[cit0015] GoB (2015), *Bangladesh Economic Review 2014*, Finance Division, Ministry of Finance, Government of Bangladesh, Dhaka, available at: http://mof.gov.bd/en/index.php?option=com_content&view=article&id=304&Itemid=1 (accessed November 14, 2015).

[cit0016] GoB (2017), *Bangladesh Economic Review 2017*, Finance Division, Ministry of Finance, Government of Bangladesh, Dhaka, available at: www.mof.gov.bd/site/page/44e399b3-d378-41aa-86ff-8c4277eb0990/BangladeshEconomicReview (accessed February 12, 2018).

[cit0017] Greene W. (2005), “Reconsidering heterogeneity in panel data estimators of the stochastic frontier model”, *Journal of Econometrics*, Vol. 126 No. 2, pp. 269–303.

[cit0018] Islam M. (2015), “Essays on development economics”, doctoral dissertation, Graduate School of Arts and Science, The Harvard University, Cambridge, MA, available at: http://citeseerx.ist.psu.edu/viewdoc/download?doi=10.1.1.985.1472&rep=rep1&type=pdf (accessed December 29, 2017).

[cit0019] Miyata S., Minot N. and Hu D. (2009), “Impact of contract farming on income: linking small farmers, packers, and supermarkets in China”, *World Development*, Vol. 37 No. 11, pp. 1781–1790.

[cit0020] MoA (2017), *Subsidy and Other Information: Subsidy on Fertilizers in the Last Seven Years*, Ministry of Agriculture, Dhaka, available at: http://moa.portal.gov.bd/sites/default/files/files/moa.portal.gov.bd/page/028b9a5c_4fe9_4341_8f9a_f2d9c4bbb62b/urea_15-11-2015.pdf (accessed December 29, 2017).

[cit0021] MoA and FAO (2013), *Master Plan for Agricultural Development in the Southern Region of Bangladesh*, Ministry of Agriculture, Government of Bangladesh and United Nations Food and Agriculture Organization, Dhaka, p. 122.

[cit0022] Mottaleb K.A. and Mohanty S. (2015), “Farm size and profitability of rice farming under rising input costs”, *Journal of Land Use Science*, Vol. 10 No. 3, pp. 243–255.

[cit0023] Mottaleb K.A. and Sonobe T. (2011), “An inquiry into the rapid growth of the garment industry in Bangladesh”, *Economic Development and Cultural Change*, Vol. 60 No. 1, pp. 67–89.

[cit0024] Otsuka K. and Hayami Y. (1988), “Theories of share tenancy: a critical survey”, *Economic Development and Cultural Change*, Vol. 37 No. 1, pp. 31–68.

[cit0025] Perveen F. (2016), *Crop Monitoring System in Bangladesh, Main Challenges, Recent Initiatives and Prospects*, Department of Agricultural Extension, Ministry of Agriculture, Bangladesh, Dhaka, available at: www.fao.org/fileadmin/templates/rap/files/meetings/2016/160524_AMIS-CM_4.1.1_Crop_Monitoring_System_in_Bangladesh_Main_Challenges_and_Recent_Initiatives.pdf (accessed December 29, 2017).

[cit0026] Rapsomanikis G. (2015), *The Economic Lives of Smallholder Farmers: An Analysis Based on Household Data from Nine Countries*, Food and Agriculture Organization of the United Nations, Rome, p. 6, available at: www.fao.org/3/a-i5251e.pdf (accessed February 14, 2018).

[cit0027] Rivera W.M., Qamar M.K. and van Crowder L. (2001), *Agricultural and Rural Extension Worldwide: Options for Institutional Reform in the Developing Countries*, Food and Agriculture Organization of the United Nations, Rome, available at: www.fao.org/tempref/docrep/fao/004/y2709e/y2709e.pdf (accessed December 29, 2017).

[cit0028] Sonobe T., Hu D. and Otsuka K. (2002), “Process of cluster formation in China: a case study of a garment town”, *Journal of Development Studies*, Vol. 39 No. 1, pp. 118–139.

[cit0029] World Bank (2017), *World Development Indicators 2017: Arable Land (Hectares per Person)*, World Bank, Washington, DC, available at: http://databank.worldbank.org/data/reports.aspx?source=world-development-indicators# (accessed December 29, 2017).

[cit0030] GoB (2016), *Bangladesh Economic Review 2016*, Finance Division, Ministry of Finance, Government of Bangladesh, Dhaka, available at: https://mof.gov.bd/en/index.php?option=com_content&view=article&id=372&Itemid=1 (accessed December 29, 2017).

